# Addressing sickness absence among adolescents and young adults: an evaluation of the Medical Advice for Sick-reported Students intervention

**DOI:** 10.1186/s12889-020-09809-9

**Published:** 2020-12-03

**Authors:** Suzanne J. van den Toren, Carmen B. Franse, Yvonne T. M. Vanneste, Rienke Bannink, Marjolein Lugtenberg, Wico C. Mulder, Marlou L. A. de Kroon, Amy van Grieken, Hein Raat

**Affiliations:** 1 Department of Public Health, Erasmus University Medical Center, P.O. Box 2040, Rotterdam, 3000 CA the Netherlands; 2 Dutch Center for Youth Health (NCJ), Utrecht, the Netherlands; 3 Center for Child and Family Rotterdam Rijnmond, Rotterdam, the Netherlands; 4 Department of Health Sciences, University Medical Center Groningen, Groningen, the Netherlands

**Keywords:** Addressing sickness absence, school absenteeism, young adults, school-based intervention; MASS intervention evaluation.

## Abstract

**Background:**

Sickness absence is associated with lower school achievements and early school leaving. The Medical Advice for Sick-reported Students (MASS) intervention is a proactive school-based intervention focused primarily on early identification and reduction of sickness absence. This study used a program evaluation framework to evaluate the MASS intervention among intermediate vocational education students and Youth Health Care professionals. Outcome indicators were primarily number of sick days, education fit, and school performance, and secondarily, seven health indicators. Process indicators were dose delivered and received, satisfaction, and experience.

**Methods:**

The MASS intervention evaluation was conducted in ten intermediate vocational education schools. Students with extensive sickness absence from school in the past three months were included in either the intervention or control condition. Students completed a baseline and a six-month follow-up self-report questionnaire. Linear and logistic regression analyses were applied. Students and Youth Health Care professionals completed an evaluation form regarding their satisfaction and experience with the intervention.

**Results:**

Participants (*n* = 200) had a mean age of 18.6 years (SD = 2.02) and 78.5% were female. The MASS intervention showed positive results on decreasing sickness absence in days (β = -1.13, 95% CI = -2.22;-0.05, *p* < 0.05) and on decreasing depressive symptoms (β = -4.11, 95% CI = -7.06;-1.17, *p* < 0.05). No effects were found for other health indicators (*p* > 0.05). A significant interaction revealed a decline in sickness absence in males (*p* < 0.05) but not in females (*p* > 0.05). Youth Health Care professionals found the application of the MASS intervention useful (*n* = 35 forms). The mean rating of students for the consultation within the MASS intervention was an 8.3 (SD = 1.3) out of 10 (*n* = 14 forms).

**Conclusions:**

Our study provides some indication that the MASS intervention has positive effects on decreasing both sickness absence and depressive symptoms among intermediate vocational education students. The Youth Health Care professionals who provided the consultation as part of the MASS intervention considered the intervention to be useful and stated that the consultation was delivered as intended in almost all cases. Students were generally satisfied with the intervention. We recommend that future research evaluates the MASS intervention in a large randomized controlled trial with a longer follow-up.

**Trial registration:**

This study was prospectively registered in the Netherlands Trial Register under number NTR5556, in October 2015.

**Supplementary Information:**

The online version contains supplementary material available at 10.1186/s12889-020-09809-9.

## Background

Addressing frequent and/or prolonged school absenteeism among adolescents and young adults is considered to be of great societal importance as it is associated with decreased school performance and increased dropout rates [1]. School absenteeism can be divided into excused absence (e.g. sickness absence) and unexcused absence (e.g. truancy) [1, 2]. Sickness absence is generally found to be more prevalent than truancy among adolescents [3–5]. In a study in the Netherlands, 40% of adolescents and young adults reported one or more sick days in the past month [4].

Another study found that more than half the cases of extensive sickness absence among adolescents were associated with problems such as psychosocial problems, sleeping difficulties, and lifestyle problems rather than related to a specific condition [6]. School-related factors, such as elevated study pressure, are also linked to sickness absence [7]. Increased sickness absence was also found to be associated with decreased mental and physical health-related quality of life, with physical complaints, and with decreased school performance [6, 8–10]. Extensive sickness absence, a negative school attitude, and lower levels of achievement are strongly associated with early school leaving [11]. Early school leavers are students of up to 23 years old who leave education and training without attaining a basic education qualification for successfully entering the labor market [12, 13]. These early school leavers are more vulnerable in terms of having lower earnings, needing government assistance, and reporting poor physical health than peers who do obtain their basic education qualification [1, 14–16]. Adolescents who leave school due to health issues have been found to be especially vulnerable [17].

Sickness absence and early school leaving might be prevented through early identification of those students who report sick in order to evaluate whether additional support is needed. The Medical Advice for Sick-reported Students (MASS) intervention is a proactive school-based intervention with the focus on addressing sickness absence and associated indicators of health. In this intervention, schools collaborate directly with the Dutch Youth Health Care system. This system offers nationwide preventive health care through anticipatory guidance for children and youth to promote growth, development, health, and well-being. The MASS intervention was initially implemented in pre-vocational education among students aged 12 to 16 years [18]. A significantly larger reduction in sickness absence was observed among students in the intervention condition receiving the MASS intervention than in the control condition. The MASS intervention has since been adapted to intermediate vocational education settings. In the Netherlands, this education is a type of upper secondary education (International Standard Classification of Education level 3), offering specialized job-oriented programs [13]. As students in intermediate vocational education are older (i.e. aged 15 years and older) than students in pre-vocational education, other factors may play a role in their reporting sick [7, 19, 20]. Furthermore, the majority of adolescents and young adults leave school when enrolled in intermediate vocational education [21, 22]. Therefore, it is important to evaluate the MASS intervention among these students.

In this study, we applied the framework for program evaluation in public health from the Centers for Disease Control and Prevention (CDC) [23] to guide the MASS intervention evaluation. In this framework, a six-step approach toward program evaluation is proposed, including describing the program (i.e. the MASS intervention in our study) and justifying the conclusions. This framework advises to evaluate outcome indicators to measure whether the program is achieving the expected effects and process indicators to measure the programs’ activities. First, we evaluated whether, at follow-up, students from intermediate vocational education with extensive sickness absence in the intervention condition have less sickness absence, a higher education fit, and higher school performance than students with extensive sickness absence who receive care as usual. In this regard, we further explored seven health indicators and hypothesize that students in the intervention condition will score better on these outcomes. Second, we evaluated the dose of the intervention delivered and received, and the satisfaction and experience of the intervention among students who received the intervention and the Youth Health Care professionals who delivered the intervention.

## Methods

### Study design

The program evaluation of the MASS intervention was conducted between December 2015 and April 2017 [24].

**Fig. 1:**
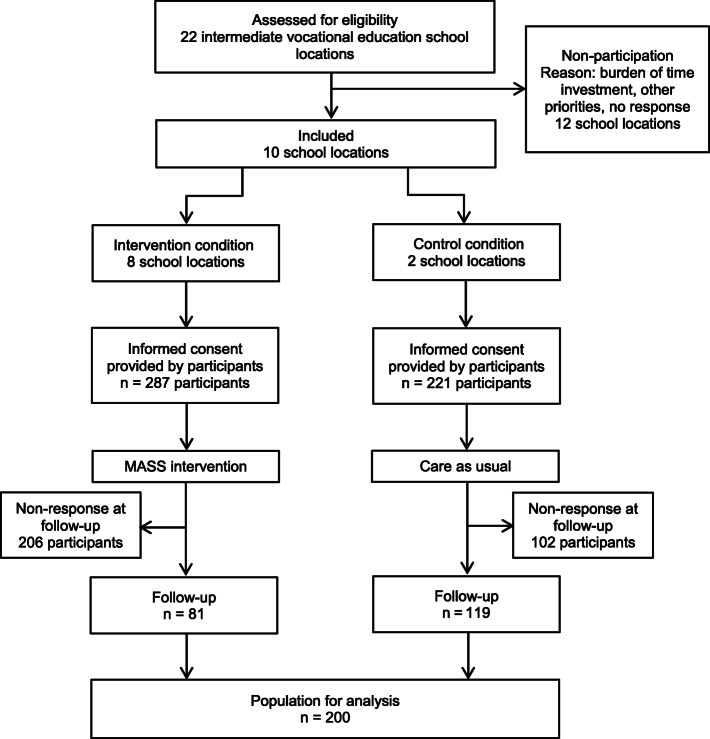
Flow chart of the study population

A total of 22 intermediate vocational education school locations were invited to participate in the study (Fig. 1). Twelve locations were not able to participate, mainly because of the anticipated time investment. Finally, eight locations in the city of Amsterdam and the regions of Utrecht and West-Brabant participated as intervention schools. These regions had implemented the MASS intervention during the course of our study as part of local policy. Two locations in the city of Rotterdam where the MASS intervention had not been implemented participated as control schools. These schools provided care as usual, which generally entailed a referral to a Youth Health Care professional on request of the student and, if possible, a consultation within the Care Advisory Team about the student [24]. Our intention was to include an equal number of students in the intervention and control condition, regardless of the number of participating schools.

The Medical Ethics Committee of the Erasmus University Medical Center Rotterdam reviewed the research proposal and gave permission to submit the results for publication in a scientific journal in the future (proposal number MEC-2015-614). This study was registered in the Netherlands Trial Register under number NTR5556, and it was reported according to the TREND guidelines [25].

### Engage stakeholders

The schools and Youth Health Care professionals who implemented the MASS intervention were included in the development and the evaluation of the intervention, as were the students who were involved in the MASS intervention [7, 24, 26, 27]. Youth Health Care professionals and researchers co-designed the participant questionnaire. Results of the current study were disseminated among schools and Youth Health Care professionals.

### Describing the MASS intervention

The activities of the MASS intervention focus on the individual level (i.e. the student) and on the school level (i.e. school staff and policy) [19, 24]. The individual-level activities entail a systematic five-step approach (see Table 1): 1) Appointed school personnel, most likely a mentor or counselor, contacts the student to address concerns about the sickness absence on the day of the sickness report. 2) A meeting between the student and appointed school personnel is arranged when the sickness absence becomes extensive (criteria predefined by each school). 3) The school refers the student to a consultation with a Youth Health Care professional when this is deemed necessary. The school explains the benefit of this consultation to the student and hands out an information folder. 4) The professional analyses in depth the reason why a student reported himself sick, and then advises on and supports reintegration into school by making an action plan with the student and his/her parents for reintegration. For this, the biopsychosocial model and the self-sufficiency matrix are used. 5) The school is responsible for monitoring the sickness absence of the student and for implementing the action plan for reintegration, if this is created. The student who reported sick is not required to go through all five steps. The next step is only taken when the school deems this necessary as a result of ongoing or increasing sickness reporting by the student or when the student requests this.

**Table 1:** Description of the key steps of the MASS intervention derived from van der Vlis et al. [22]

Step	Description
**1**	The school contacts the student who reports sick and asks about the context of the sickness report and condition of the student.
**2**	The school organizes a meeting with the student in case of extensive sickness absence (criteria predefined by each individual school). Parents are also invited when the student is younger than 18 years of age.
**3**	The school refers the student to a consultation with a Youth Health Care professional when this is deemed necessary and explains the benefit of this consultation to the student.
**4**	A consultation is organized by the regional Youth Health Care organization with the Youth Health Care professional and the student (and parents if the student is younger than 18 years old). Together they conduct a problem analysis, define the underlying problems and causes of the absence, using the biopsychosocial model and the self-sufficiency matrix. The possibilities of preventing recurrence of the absence and treatment are discussed and an action plan for reintegration is created, which is communicated to the school.
**5**	The school is responsible for monitoring the sickness absence of the student and school-related implementation of the reintegration plan, if created.

The school-level activities entail agreements at policy level on how to actively monitor students’ absences, i.e. approach students with extensive sickness absence via personal contact, request a consultation with the Youth Health Care professional, and arrange a follow-up for each student in the Care Advisory Team. In this team, Youth Health Care professionals, teachers, and other personnel who are concerned with students’ health meet regularly to raise concerns about students and offer care to them. The MASS intervention is developed by following the intervention mapping approach [28, 29]. Additionally, the intervention is based on Veerman’s decision-making model, which was derived from occupational sickness absence. This model first considers the absence necessity (feeling sick) and then deliberates on weighing the pros and cons of reporting sick [30].

### Participants and data collection

In both study conditions, students were included if they had ‘extensive’ sickness absence in the past three months (i.e. reporting sick at least four times or more than six consecutive school days in twelve school weeks).

To meet the wishes and ability of the schools to take part, two procedures were followed to select participants. At eight schools (i.e. seven intervention and one control school), an employee at the schools selected and invited students to participate if they met the inclusion criteria. At two schools (i.e. one intervention and one control school), whole classes were invited, and the involved researcher selected students who met the inclusion criteria afterward.

All selected students received an information letter and leaflet with information about the study from an employee at the schools. If they agreed to participate, they were asked to provide written consent. This consent form was attached to the baseline questionnaire. If eligible students were younger than 18 years old, their parents also received an information letter and brochure about the study, explaining that, if desired, they could object to their child’s participation. Figure 1 presents the flow of participants through the study.

### Measurements

Data were collected among students at baseline and at six-month post-baseline using a self-report questionnaire [24].

#### Program evaluation indicators

##### Primary outcome indicators

Sickness absence was measured with the item: “How many days in the past eight school weeks did you stay home from school because you were sick? (Do not count holidays)” [31].

Education fit was measured with the item: “Do you think this is a good education fit for you?”; answer options were dichotomized for analysis purposes: ‘yes’ (‘yes’ and ‘a little’) and ‘no’ (‘I do not know’, ‘not really’, and ‘no’) [31].

School performance was measured with the item: “How do you think your teacher estimates your school performance compared to other classmates?”; answer options were dichotomized for analysis purposes: ‘above average’ (‘very good’ and ‘good’) and ‘average or less’ (‘average’, ‘less than average’, and ‘bad’) [32].

##### Secondary outcome indicators

The Center for Epidemiologic Studies Depression scale (CES-D) was used to measure the scale of depressive symptoms. The CES-D is a validated 20-item scale covering the main components of depressive symptoms such as depressed mood, guilt, and feelings of inferiority (reported Cronbach’s alpha coefficients across studies ranged from 0.85–0.90) [33]. The frequency of experiencing these symptoms was scored on a 4-point scale (answer categories ranging from ‘always’ to ‘hardly ever’). These scores are summed (range 0–60), with higher scores indicating higher levels of depressive symptoms [34–36].

The validated 12-item Short-Form Health Survey (SF-12) was used to measure health-related quality of life. The SF-12 includes 12 items from which the Physical Component Summary (PCS-12) score and the Mental Component Summary (MCS-12) score were calculated (test-retest correlations of 0.89 and 0.76 were previously observed) [37]. These scores ranged from 0 to 100, with higher scores indicating better health-related quality of life.

Truancy was measured with the item: “Have you been truanting in the past four weeks?”(answer categories ranged from ‘no’ to ‘more than 20 hours’) [31]. For analysis purposes, truancy in the past 4 weeks was dichotomized into ‘yes’ and ‘no’.

Financial and housing problems were measured with two corresponding items from the adapted and validated Self-Sufficiency Matrix (SSM-D) (reported Cronbach’s alpha coefficients were 0.85 and 0.89) [38, 39]. This matrix measures self-sufficiency with answer categories ranging from ‘no problems’ to ‘many problems’. For analysis purposes, the answer options were dichotomized into ‘self-sufficient’ and ‘not to barely self-sufficient’.

Criminal behaviors were measured with eleven items asking about criminal behaviors in the past six months, e.g. stealing something worth 5 euro and having been questioned at the police station (answer categories ranged from ‘never’ to ‘six or more times’). For analysis purposes, the answer options were dichotomized into ‘no criminal behaviors’ and ‘at least one criminal behavior’ in the past six months.

Potential confounders in assessing the primary and secondary outcome indicators were socio-demographic characteristics of the students, which included gender, age, intermediate vocational educational level (higher level 4 versus lower levels 1–3), ethnic background (Dutch versus non-Dutch according to the classification of Statistics Netherlands [40]), and living situation. Other potential confounders were binge drinking, cigarette smoking, and cannabis use. These were all dichotomized into ‘yes’ and ‘no’ [31].

##### Process indicators

Specific process indicators that we evaluated included dose delivered and received, satisfaction, and experience [41]. The Youth Health Care professionals who provided the intervention and the students who had a consultation with the professional filled out an evaluation form after the consultation according to step four of the intervention (see Table 1). The extent to which the professional and the school worked in accordance with the MASS intervention (dose delivered) was measured with four statements (see Table 5). Answer options were ‘yes’ or ‘no’. Professionals’ satisfaction and experience with the intervention were measured with three items regarding the usefulness of the application of the MASS intervention (see Table 5). Answer options ranged from ‘very useful’ to ‘not useful’.

Students’ satisfaction and experience with the consultation were measured with nine statements (e.g. ‘I felt I was taken seriously by the Youth Health Care professional’). Answer options ranged from ‘strongly disagree’ to ‘strongly agree’. Furthermore, the students gave a grade from one to ten for their satisfaction with the consultation with the Youth Health Care professional.

We evaluated the dose received in the second and fourth steps of the MASS intervention in the main self-report questionnaire at follow-up by asking whether students had a meeting with the school regarding their sickness absence and whether they had a consultation with a health care professional regarding their sickness absence.

### Statistical analysis

Descriptive statistics were used to describe the characteristics and outcomes of students in both study conditions. Differences between the intervention condition and the control condition at baseline were tested by chi-square tests (for categorical variables) and independent sample *t*-tests (for continuous variables).

The primary and secondary outcome indicators were investigated using linear (for continuous variables) and logistic (for categorical variables) regression analyses regarding primary and secondary outcome indicators. In the first model, each outcome was predicted with the study condition (intervention and control) and corresponding baseline value as predicting variables (crude model). In the second model, further adjustments to the crude model were made by adding potential confounders (adjusted model). The selection of these confounders was based on the literature and significant differences between the study conditions at baseline for socio-demographic and lifestyle behaviors. As such, gender and intermediate vocational education level were added as confounders. *p* < 0.05 was considered statistically significant.

Subsequently, we explored whether gender, ethnic background, and the presence of a clinically relevant number of depressive symptoms (yes/no) moderated the effect of the intervention [24]. This was done by adding a study condition*possible moderator interaction term to the regression analyses for primary outcome indicators. If the interaction term was significant at *p* < 0.10, a stratified analysis was conducted.

The intraclass correlation coefficient (ICC) was calculated to consider the potential variance in sickness absence explained by the clustering of schools. The estimated ICC was equal to zero (ICC < 0.001); therefore, no adjustment for school was performed in subsequent analyses.

Descriptive statistics were used to analyse the evaluation forms filled out by students who received the intervention and Youth Health Care professionals who provided the intervention.

All analyses were performed using SPSS version 25 for Windows (IBM Corp. Released 2017. IBM SPSS Statistics for Windows, Version 25.0, IBM Corp., Armonk, NY, USA).

## Results

### Participant’ characteristics

In total, 287 participants were included in the intervention condition, and 221 participants were included in the control condition at baseline. At six-month follow-up, 81 participants in the intervention condition (28.2%) and 119 participants in the control condition (53.9%) completed the questionnaire (Fig. 1). Reasons for not participating in the follow-up measurement were reported as an unwillingness to participate and the relocation of the participant.

We compared participants who completed both the baseline and follow-up questionnaire with participants who did not reply to the follow-up questionnaire. Those who did not reply to the follow-up questionnaire were more often male (*p* < 0.001), were lower educated (*p* < 0.001), were more often classified as non-Dutch (*p* < 0.05), and had a worse education fit (*p* < 0.05) than participants included in both measurements. Stratified by study condition, those who did not reply to the follow-up questionnaire in the intervention condition were more often male (*p* < 0.001), were lower educated (*p <* 0.05), and were more often classified as non-Dutch (*p* < 0.05) than participants included in both measurements. Those who did not reply to the follow-up questionnaire in the control condition were more often male (*p <* 0.05) and were more often classified as non-Dutch (*p* < 0.05) than participants included in the control condition at follow-up (see Additional file 1, Table A1).

Table 2 presents an overview of the socio-demographic and lifestyle characteristics of the study population at baseline. The mean age was 18.6 years (SD 2.1), and 78.5% were female. Participants in the intervention condition attended lower education levels than participants in the control condition (*p* = 0.01). No other differences were observed (*p* > 0.05).

**Table 2:** Socio-demographic and lifestyle characteristics of the intervention and control condition at baseline (*N* = 200)

		Total	Intervention condition	Control Condition	*p*-value
		*N* = 200	*n* = 81	*n* = 119	
**Socio-demographic characteristics**					
Age in years, mean (SD)	[0]	18.6 (2.0)	18.6 (2.1)	18.6 (2.0)	.941
Female gender, n (%)	[0]	157 (78.5)	60 (74.1)	97 (81.5)	.209
Intermediate vocational education level 4, n (%)^a^	[8]	153 (79.7)	56 (70.9)	97 (85.8)	**.011**
Dutch ethnic background, n (%)	[4]	152 (77.6)	61 (77.2)	91 (77.8)	.926
Living at home with caretaker, n (%)	[1]	177 (88.9)	71 (88.8)	106 (89.1)	.943
**Lifestyle characteristics**					
Current smoking, n (%)	[9]	50 (26.2)	24 (30.0)	26 (23.4)	.308
Binge drinking in past 4 weeks, n (%)^b^	[7]	86 (44.6)	39 (48.8)	47 (41.6)	.324
Cannabis use in past 4 weeks, n (%)	[10]	29 (15.3)	12 (15.0)	17 (15.5)	.931

[number of missing answers]. Bold numbers indicate statistical significance (*p* < 0.05) between the intervention condition and the control condition, calculated using an independent-samples t-test (continuous variables) or a chi-square test (categorical variables)

^a^Intermediate vocational education consists of four levels: level 1 assistant training; level 2 basic vocational training; level 3 vocational training; level 4 middle-management training. Level 4 is considered the highest level

^b^Binge drinking was defined as consuming 5 or more alcoholic drinks on one occasion

### Primary and secondary outcome indicators

Table 3 shows the differences between the study conditions at both time measurements for primary outcome indicators. At baseline, the number of sick days in the past 8 weeks was higher in the intervention condition than in the control condition (*p* = 0.028). At follow-up, education fit was higher in the intervention condition than in the control condition (*p* = 0.040).

**Table 3:** Differences between intervention and control condition at baseline and follow-up for primary outcomes

Primary outcomes	Baseline				Follow-up			
	Total	Intervention condition	Control condition	*p*-value	Total	Intervention condition	Control condition	*p*-value
Days of sickness absence in past 8 weeks, mean (SD)	6.0 (6.6)	7.2 (6.4)	5.1 (6.6)	**.028**	3.0 (3.7)	2.9 (3.4)	3.1 (3.9)	.803
Education fit, n yes/a bit (%)^a^	167 (87.4)	68 (86.1)	99 (88.4)	.634	156 (88.1)	68 (94.4)	88 (83.8)	**.032**
School performance, n above average/average (%)^b^	124 (64.6)	49 (61.3)	75 (67.0)	.414	140 (79.1)	59 (81.9)	81 (77.1)	.440

Note: bold numbers indicate statistical significance (*p* < 0.05) between the intervention condition and the control condition, calculated using an independent-samples t-test (continuous variables) or a chi-square test (categorical variables)

^a^Measured on a 5-point Likert scale, dichotomized into ‘yes’ (i.e. ‘yes’ and ‘a bit’) and ‘no’ (i.e. ‘I do not know-no’)

^b^Measured on a 5-point Likert scale, dichotomized into ‘good’ (i.e. ‘very good’ and ‘good’) and ‘not good’ (i.e. ‘average and less’)

Table A2 in Additional file 2 shows the differences between the study conditions at both time measurements for secondary outcome indicators. At follow-up, the intervention condition had fewer depressive symptoms (*p* = 0.003) and a higher mental health-related quality of life (*p* = 0.012) than the control condition. No other differences were observed (*p* > 0.05).

Table 4 shows the association of study condition with primary outcome indicators. The crude model showed an increase in education fit (OR = 4.37, 95% CI = 1.25; 15.25), which was no longer visible in the adjusted model. The adjusted model showed an average decrease of 1.13 sick days in the past 8 weeks (β = - 1.13, 95% CI = -2.22;-0.05, *p* = 0.04) among students in the intervention condition compared with those in the control condition.

**Table 4:** The association of study condition with primary outcome measures

Primary outcomes	Crude model^a^	Adjusted model^b^
	Intervention vs control condition	Intervention vs control condition
	B (95% CI)	B (95% CI)
Days of sickness absence in past 8 weeks	-0.71 (−1.77;0.35)	**-1.13 (-2.22;-0.05)**
	OR (95% CI)	OR (95% CI)
Education fit (yes/a bit)^c^	**4.37 (1.25; 15.25)**	3.61 (0.98;13.31)
School performance (very good/good)^d^	1.81 (0.81; 4.07)	1.77 (0.78; 4.03)

Note: bold numbers indicate statistical significance (*p* < 0.05) between the intervention condition and the control condition, calculated using linear or logistic regression models with the control condition as reference

^a^Model of follow-up score with correction for corresponding baseline score, without correction for confounders

^b^Model of follow-up score with correction for corresponding baseline score, intermediate vocational education level and gender

^c^Measured on a 5-point Likert scale, dichotomized into ‘yes’ (i.e. ‘yes’ and ‘a bit’) and ‘no’ (i.e. ‘I do not know-no’)

^d^Measured on a 5-point Likert scale, dichotomized into ‘good’ (i.e. ‘very good’ and ‘good’) and ‘not good’ (i.e. ‘average and less’)

Missings: Baseline days of sickness absence in past eight weeks = 8, follow-up days of sickness absence in past eight weeks = 20; baseline education fit = 9, follow-up education fit = 23; Baseline school performance = 8, follow-up school performance = 23; intermediate vocational education level = 8; gender = 0

Table A3 in Additional file 3 shows the association of study condition with secondary outcome indicators. Participants in the intervention condition showed a decrease of depressive symptoms compared with those in the control condition (β = - 4.11, 95% CI = -7.06;-1.17, *p* = 0.01). No other differences were observed (*p* > 0.05).

A significant interaction was found between study condition and gender (*p* = 0.002) on sickness absence. The stratified analyses revealed a significant decline in sickness absence in males (β = - 4.3, 95% CI = -6.5;-2.1, *p* < 0.001) and not in females (β = - 0.18, 95% CI = -1.4;1.05, p = 0.775).

### Process indicators

We received a total of 35 evaluation forms from Youth Health Care professionals (Table 5). In 34/35 cases, the professionals experienced the application of the MASS intervention as very useful or useful. In 31/35 cases, the professionals made a reintegration plan with the student. In 35/35 cases, they communicated the agreements originating from the consultation with the student with the school staff.

**Table 5:** Evaluation of the consultation within the MASS intervention by Youth Health Care professionals

Process indicator	Statement	
Satisfaction with MASS intervention		% very useful and useful (n/total n)
	How did you experience the application of the MASS intervention?	97.1 (34/35)
	How did you experience the application of the biopsychosocial model?	87.9 (29/33)
	How did you experience the application of the self-sufficiency matrix?	74.3 (26/35)
Use of MASS intervention		% yes (n/total n)
	Did school contact the student in response to the sickness absence?	100.0 (29/29)
	Did the school explain the added value of the consultation with the Youth Health Care professional?	96.6 (28/29)
	Did you make a reintegration plan with the student?	88.6 (31/35)
	Did you communicate the agreements you made with the student to school?	100.0 (35/35)

We received a total of 14 evaluation forms from students. Almost all students (12/14) answered that they (strongly) agreed with the statement that the Youth Health Care professional had taken them seriously, that they trusted this professional, and that the professional listened to them. The majority (10/14) agreed with the statement ‘I dared to ask the Youth Health Care professional questions’. The mean rating for the consultation was an 8.3 (SD = 1.3) out of 10.

In the follow-up questionnaire, around half of the students in the intervention condition indicated that they had received the second step of the intervention (51.9%). A little less than 20% of students in the intervention condition indicated that they had received the fourth step of the intervention (17.3%).

## Discussion

In this study, a program evaluation framework was applied to evaluate the MASS intervention. A decrease in sickness absence and depressive symptoms was found among participants in the intervention condition at six-month follow-up compared with those in the control condition. The Youth Health Care professionals who provided the consultation as part of the MASS intervention considered the intervention to be useful, and students who had followed all steps of the intervention appreciated the consultation that was part of the intervention.

In accordance with step five of the program evaluation framework (i.e. justify conclusions), the results will be interpreted alongside the literature. Our finding that a decrease in sickness absence was found in those in the intervention condition at six-month follow-up, compared with those in the control condition is in line with a previous study evaluating the MASS intervention at pre-vocational education [18]. A meta-analysis by Tanner-Smith et al. also found that vocationally oriented programs showed promise in reducing school absenteeism [42]. As suggested by previous research [1, 43], the reduction in sickness absence may be attributed to both the increased monitoring of sickness absence and to the systematic collaboration between schools and health personnel. Additionally, factors such as an anonymous sickness reporting procedure at school, or the lack of a reaction to the sickness report by the school might make it ‘easier’ for students to report sick [26]. The MASS intervention actively monitors and systematically handles sickness absence from the first day of absence. Moreover, another reason for the reduction in sickness absence could be that adolescents with a record of high school absenteeism may not have timely contact with health care professionals [43]. For these adolescents, the MASS intervention could be the initiation of contact with a health care professional who can help with the underlying reasons for their sickness absence. However, we learned that relatively few participants go through step four of the intervention (i.e. the consultation with the Youth Health Care professional). It is unclear whether this is primarily a result of reduced sickness reporting prior to step four or if students perceive obstacles in attending the consultation. Suggestions to further improve the intervention and to make the intervention more approachable for students entail the use of digital tailored messages to students with extensive sickness absence or to develop a MASS app to meet the growing need for online support [44]. Indeed, phone apps have been found to improve physical and mental health outcomes [45].

The positive finding that a larger reduction of depressive symptoms was found in the intervention condition might be a result of the specific attention given by school personnel and the Youth Health Care professional to the students’ mental and physical health. Depression has been found to be associated with sickness absence [1, 46]; therefore, it may be important to address depressive symptoms and mental health when addressing sickness absence.

The number of participants who did not reply to the follow-up questionnaire was relatively large, especially in the intervention condition. MASS is implemented in schools as a whole (implementing sickness policy in schools and requiring the school to contact a student after a first sick-report). As such, all students with sickness absence from intervention schools are assumed to be exposed to the intervention. Analyses showed that those who did not reply to the follow-up questionnaire were more often male, lower educated, more often classified as non-Dutch, and had a lower education fit. Although we included education level and gender as confounders and adjusted for corresponding baseline values in our analyses, it is possible that this selective non-response to follow-up led to an underestimation or overestimation of the results. This was especially the case when more motivated students participated in the follow-up measurement, which may have led to more positive results. We should therefore be careful with interpreting and generalizing the results. Taking this into account, we recommend replicating this study in large and varied populations. In the future, telephone reminders to non-respondents and incentives of interest to participants might contribute to a higher response to questionnaires [47]. Although non-response to the follow-up questionnaire was high, the results provide preliminary information for schools and health personnel who wish to reduce sickness absence from school. As early school leaving is a main consequence of sickness absence and is highest among intermediate vocational education students, our results indicate that the MASS intervention may contribute to the prevention of early school leaving, which, in turn, is an important outcome for public health.

Explorative analyses showed that the significant decrease in sickness absence was only observed in males. This was in accordance with a meta-analysis by Tanner-Smith et al. that found that positive effects of interventions on school absenteeism were predominantly detected in males [42]. An explanation for this might be that males are found less likely to seek help or delay help-seeking, for example, for depression [48, 49]; however, in the MASS intervention, the help was offered to them timely and proactively. Another explanation might be found in the higher non-response to the follow-up questionnaire among males in the current study. Here, possibly more males with higher sickness absence did not reply to the follow-up questionnaire. Future research should study this possibility.

According to the Youth Health Care professionals, the consultation (step 4 of the MASS intervention) was delivered as intended in almost all cases, as was the intervention in general. In a small portion of cases, however, the integration plan was not delivered. Since the integration plan for a student to join classes again is an important part of the MASS intervention, future research should address whether the delivery of the integration plan can be optimized. In almost all cases, the Youth Health Care professionals experienced the application of the MASS intervention to be useful. Despite this positive response, it might be possible to improve the MASS intervention by further evaluating the usefulness of the self-sufficiency matrix as part of the consultation. Students were generally satisfied with the intervention and felt that they were taken seriously by the Youth Health Care professional in almost all cases. Being treated seriously has been shown to be an important aspect of preventing sickness reporting according to students in a previous study [26]. Some students indicated that they did not dare to ask questions to this professional during the consultation. Previous research has showed that a bond of trust between youth and a health care professional (e.g. a general practitioner) is often lacking [50], which might result in a barrier against students asking questions. Perhaps, as a means of resolving this issue, Youth Health Care professionals could be better trained to emphasize the possibility of students to ask questions and to invest in a bond of trust.

No significant positive results of the MASS intervention were found for the other primary and secondary outcome indicators, such as school performance, criminal behaviors, or health-related quality of life. Although students’ education fit and school performance at follow-up were higher in the intervention condition than in the control condition, the improvement was not significantly different between the students in both conditions. There could be two reasons for this: first, these outcomes may be more indirectly affected by the intervention; and second, changes in behavior could take more than the six months follow-up time in our study. We therefore recommend that future studies evaluate the MASS intervention in a large randomized controlled trial and with a longer follow-up time of 1 to 2 years.

### Study limitations

Some methodological considerations need to be discussed. Firstly, while the number of students that was included at baseline in the intervention and in the control condition was similar, eight schools contributed to the intervention condition and two schools to the control condition. For future evaluation studies, we recommend balancing the intervention and control condition by applying the design of a cluster-randomized trial. Secondly, the validity of the measurements needs to be considered. Sickness absence was self-reported by the students. It might be that students do not recall their sickness absence or that they might give socially desirable answers. For a subgroup of 44 participants in this study, additional school registry data on sickness absence were available. The correlation between sickness absence according to the school registry data and according to the self-reported data was 0.71 (*p* < 0.001; data not shown). For future studies, we recommend using school registry data regarding sickness absence. The measurement of school performance by student self-report was validated by Felder-Puig et al. in a comparison of students self-report with the students’ grades at school. The study showed that the self-report of school performance by students can distinguish groups of respondents that obtain good grades at school from those that do not [32]. For future studies, we recommend obtaining objective information on school performance in terms of students passing on to the next year or obtaining a diploma; for that, a study with a longer follow-up time is required. Thirdly, in our study, at six-month follow-up, the number of participants in the intervention condition is much lower than the number of participants in the control condition. Therefore, the results should be interpreted with caution.

## Conclusion

In conclusion, our study provides some indication that the MASS intervention has positive results in decreasing both sickness absence and depressive symptoms among intermediate vocational education students. However, especially in the intervention condition and among male students, there was a high percentage of non-response to the follow-up questionnaire. The Youth Health Care professionals who provided the consultation as part of the MASS intervention considered the intervention to be useful and stated that the consultation was delivered as intended in almost all cases. Students were generally satisfied with the intervention. We recommend that future research evaluate the MASS intervention in a large randomized controlled trial with a longer follow-up period.

## Supplementary Information

**Additional file 1: Table A1.** Non-response to follow-up analysis on socio-demographic and lifestyle characteristics (*N* = 508). Table A1 in Additional file 1 shows the comparison of participants who completed both the baseline and follow-up questionnaire with participants who did not reply to the follow-up questionnaire. Those who did not reply to the follow-up questionnaire were more often male (*p* < 0.001), were lower educated (*p* < 0.001), were more often classified as non-Dutch (*p* < 0.05), and had a worse education fit (*p* < 0.05) than participants included in both measurements. Stratified by study condition, those who did not reply to the follow-up questionnaire in the intervention condition were more often male (*p* < 0.001), were lower educated (*p <* 0.05), and were more often classified as non-Dutch (*p* < 0.05) than participants included in both measurements. Those who did not reply to the follow-up questionnaire in the control condition were more often male (*p <* 0.05) and were more often classified as non-Dutch (*p* < 0.05) than participants included in the control condition at follow-up.

**Additional file 2: Table A2.** Differences between intervention and control condition at baseline and follow-up for secondary outcomes (*N* = 200). Table A2 in Additional file 2 shows the differences between the study conditions at both time measurements for secondary outcome indicators. At follow-up, the intervention condition had fewer depressive symptoms (*p* = 0.003) and a higher mental health-related quality of life (*p* = 0.012) than the control condition. No other differences were observed (*p* > 0.05).

**Additional file 3: Table A3.** The association of study condition with secondary outcome measures. Table A3 in Additional file 3 shows the association of study condition with secondary outcome indicators. Participants in the intervention condition showed a decrease of depressive symptoms compared with those in the control condition (β = − 4.11, 95% CI = -7.06;-1.17, *p* = 0.01). No other differences were observed (*p* > 0.05).

## Data Availability

Data are available upon reasonable request by contacting the corresponding author Hein Raat (h.raat@erasmusmc.nl).

## Abbreviations

MASS: Medical Advice for Sick-reported Students
SD: Standard deviation
β: Beta coefficient
CES-D: Center for Epidemiologic Studies Depression scale
SF-12: 12-item Short-Form Health Survey
PCS: Physical Component Summary
MCS: Mental Component Summary
OR: Odds ratio
CI: Confidence interval
ICC: Intraclass correlation coefficient
