# Supplementary material for: Addressing sickness absence among adolescents and young adults: an evaluation of the Medical Advice for Sick-reported Students intervention
Source: BMC Public Health. 2020 Dec 3;20:1851. doi: 10.1186/s12889-020-09809-9 (PMC7713334; doi:10.1186/s12889-020-09809-9)
Supplement: Supplementary file 1 — Additional file 1: Table A1. Non-response to follow-up analysis on socio-demographic and lifestyle characteristics (N = 508). Table A1 in Additional file 1 shows the comparison of participants who completed both the baseline and follow-up questionnaire with participants who did not reply to the follow-up questionnaire. Those who did not reply to the follow-up questionnaire were more often male (p < 0.001), were lower educated (p < 0.001), were more often classified as non-Dutch (p < 0.05), and had a worse education fit (p < 0.05) than participants included in both measurements. Stratified by study condition, those who did not reply to the follow-up questionnaire in the intervention condition were more often male (p < 0.001), were lower educated (p < 0.05), and were more often classified as non-Dutch (p < 0.05) than participants included in both measurements. Those who did not reply to the follow-up questionnaire in the control condition were more often male (p < 0.05) and were more often classified as non-Dutch (p < 0.05) than participants included in the control condition at follow-up. [file 12889_2020_9809_MOESM1_ESM.docx]

| **Table A1.** Non-response to follow-up analyses on socio-demographic and lifestyle characteristics (N=508) | | | | | | | |
| --- | --- | --- | --- | --- | --- | --- | --- |
|  |  | **Total (N=508)** | | **Intervention condition (n=287)** | | **Control condition (n=221)** | |
|  |  | **Population for analyses**  **n=200** | **Lost to follow-up**  **n=308** | **Population for analyses**  **n=81** | **Lost to follow-up**  **n=206** | **Population for analyses**  **n=119** | **Lost to follow-up**  **n=102** |
| **Socio-demographic characteristics** |  |  |  |  |  |  |  |
| Age in years, mean (SD) | [3] | 18.6 (2.0) | 18.7 (2.3) | 18.6 (2.1) | 18.7 (2.4) | 18.6 (2.0) | 18.7 (2.1) |
| Female gender, % | [1] | **78.5**** | **56.0**** | **74.1**** | **49.5**** | **81.5*** | **69.3*** |
| Intermediate vocational education level 4, %^1^ | [25] | **79.7**** | **64.6**** | **70.9*** | **56.6*** | 85.8 | 81.7 |
| Dutch ehtnic background, % | [9] | **77.6*** | **64.7*** | **77.2*** | **64.4*** | **77.8*** | **65.3*** |
| Living at home with caretaker, % | [3] | 88.9 | 85.3 | 88.8 | 85.8 | 89.1 | 84.3 |
| **Primary outcomes** |  |  |  |  |  |  |  |
| Days of sickness absence in past 8 weeks, mean (SD) | [35] | 6.0 (6.6) | 6.8 (7.1) | 7.2 (6.4) | 7.3 (7.4) | 5.1 (6.6) | 5.9 (6.2) |
| Good ‘education fit’, %^2^ | [22] | **87.0*** | **78.6*** | 86.1 | 76.2 | 87.6 | 83.7 |
| Above average school performance, %^3^ | [23] | 64.6 | 56.3 | 61.3 | 57.2 | 67.0 | 54.3 |
| Note: [number of missing answers]. Bold numbers indicate statistical significance (*p*<0.05) between the population for analyses and lost to follow-up, calculated using an independent-samples t-test (continuous variables) or a chi-square test (categorical variables). Asterisks indicate significance level: * *p*<0.05, ** *p*<0.001.  ^1^ Intermediate vocational education consists of four levels: level 1 assistant training; level 2 basic vocational training; level 3 vocational training; level 4 middle-management training. Level 4 is considered the highest level.  ^2^ Measured on a 5-point Likert scale, dichotomized into ‘yes’ (i.e. ‘yes’ and ‘a bit’) and ‘no’ (i.e. ‘I do not know-no’)  ^3^ Measured on a 5-point Likert scale, dichotomized into ‘good’ (i.e. ‘very good’ and ‘good’) and ‘not good’ (‘average and less’) | | | | | | | |
