# Supplementary material for: Addressing sickness absence among adolescents and young adults: an evaluation of the Medical Advice for Sick-reported Students intervention
Source: BMC Public Health. 2020 Dec 3;20:1851. doi: 10.1186/s12889-020-09809-9 (PMC7713334; doi:10.1186/s12889-020-09809-9)
Supplement: Supplementary file 2 — Additional file 2: Table A2. Differences between intervention and control condition at baseline and follow-up for secondary outcomes (N = 200). Table A2 in Additional file 2 shows the differences between the study conditions at both time measurements for secondary outcome indicators. At follow-up, the intervention condition had fewer depressive symptoms (p = 0.003) and a higher mental health-related quality of life (p = 0.012) than the control condition. No other differences were observed (p > 0.05). [file 12889_2020_9809_MOESM2_ESM.docx]

| **Table A2.** Differences between intervention and control condition at baseline and follow-up for secondary outcomes (N=200). | | | | | | | | |
| --- | --- | --- | --- | --- | --- | --- | --- | --- |
|  | Baseline | | | | Follow-up | | | |
| Secondary outcomes | Total | Intervention condition | Control condition | *p*-value | Total | Intervention condition | Control condition | *p*-value |
|  |  |  |  |  |  |  |  |  |
| Depressive symptoms, mean (SD)^1^ | 15.72 (12.00) | 14.08 (12.18) | 16.86 (11.79) | .111 | 15.49 (12.81) | 12.10 (11.50) | 17.76 (13.18) | **.003** |
| Physical HRQOL,  mean (SD)^2^ | 49.80 (10.99) | 50.24 (10.19) | 49.49 (11.55) | .642 | 50.40 (10.22) | 50.57 (9.55) | 50.28 (10.69) | .854 |
| Mental HRQOL, mean (SD)^3^ | 44.29 (14.30) | 46.39 (14.19) | 42.85 (14.26) | .089 | 44.79 (14.01) | 47.93 (13.08) | 42.73 (14.26) | **.012** |
| No truancy in past 4 weeks, n (%) | 123 (63.73) | 56 (70.00) | 67 (59.29) | .127 | 124 (71.26) | 53 (74.65) | 71 (68.93) | .413 |
| No financial problems,  n (%)^4^ | 142 (73.20) | 61 (75.31) | 81 (71.68) | .574 | 130 (72.22) | 58 (78.38) | 72 (67.92) | .123 |
| No housing problems, n (%)^4^ | 180 (92.78) | 77 (95.06) | 103 (91.15) | .299 | 171 (95.00) | 72 (97.30) | 99 (93.40) | .237 |
| No criminal behavior, n (%)^5^ | 175 (92.11) | 71 (92.21) | 104 (92.04) | .965 | 157 (94.01) | 62 (93.04) | 95 (94.06) | .975 |
| Note: bold numbers indicate statistical significance (*p*<0.05) between the intervention condition and the control condition, calculated using an independent-samples t-test (continuous variables) or a chi-square test (categorical variables).  ^1^ Number of depressive symptoms as measured with the CES-D, a higher score indicates higher levels of depression symptoms (range 0-60)  ^2^ Physical health-related quality of life as measured with the Short Form-12 health survey, a higher score indicates a better quality of life (range 0–100).  ^3^ Mental health-related quality of life, as measured with the Short Form-12 health survey, a higher score indicates a better quality of life (range 0–100).  ^4^ As measured with the adapted Dutch version of the self-sufficiency matrix, on a five point Likert scale that was dichotomized  ^5^ Small and serious criminal behaviors in the past 6 months, dichotomized into never and at least one. | | | | | | | | |
