# Supplementary material for: Addressing sickness absence among adolescents and young adults: an evaluation of the Medical Advice for Sick-reported Students intervention
Source: BMC Public Health. 2020 Dec 3;20:1851. doi: 10.1186/s12889-020-09809-9 (PMC7713334; doi:10.1186/s12889-020-09809-9)
Supplement: Supplementary file 3 — Additional file 3: Table A3. The association of study condition with secondary outcome measures. Table A3 in Additional file 3 shows the association of study condition with secondary outcome indicators. Participants in the intervention condition showed a decrease of depressive symptoms compared with those in the control condition (β = − 4.11, 95% CI = -7.06;-1.17, p = 0.01). No other differences were observed (p > 0.05). [file 12889_2020_9809_MOESM3_ESM.docx]

| **Table A3.** The association of study condition with secondary outcome measures. | | |
| --- | --- | --- |
|  | Crude model^1^ | Adjusted model^2^ |
| Secondary outcomes | Intervention vs control condition | Intervention vs control condition |
|  | B (95% CI) | B (95% CI) |
| Depressive symptoms^3^ | **-4.03 (-6.85; -1.21)** | **-4.11 (-7.06;-1.17)** |
| Physical HRQOL^4^ | -0.21 (-2.78; 2.37) | 0.23 (-2.44;2.90) |
| Mental HRQOL^5^ | 3.46 (-0.13;7.05) | 2.69 (-0.93;6.32) |
|  | OR (95% CI) | OR (95% CI) |
| No truancy in past 4 weeks | 1.36 (0.67; 2.77) | 1.33 (0.64; 2.78) |
| No financial problems^6^ | 1.81 (0.84; 3.93) | 2.20 (0.98; 4.94) |
| No housing problems^6^ | 1.79 (0.30; 10.57) | 1.96 (0.32; 11.98) |
| No criminal behavior^7^ | 1.16 (0.27; 5.07) | 1.29 (0.29; 5.82) |
| Note: bold numbers indicate a statistical significance (*p*<0.05) between the intervention condition and the control condition, calculated using linear or logistic regression models with the control condition as reference.  ^1^ Model of follow-up score with correction for corresponding baseline score, without correction for confounders.  ^2^ Model of follow-up score corrected for corresponding baseline score, intermediate vocational education level and gender.  ^3^ Number of depressive symptoms as measured with the CES-D, a higher score indicates higher levels of depression symptoms (range 0-60)  ^4^ Physical health-related quality of life as measured with the Short Form-12 health survey, a higher score indicates a better quality of life (range 0–100).  ^5^ Mental health-related quality of life, as measured with the Short Form-12 health survey, a higher score indicates a better quality of life (range 0–100).  ^6^ As measured with the adapted Dutch version of the self-sufficiency matrix, on a five point Likert scale that was dichotomized  ^7^Small and serious criminal behaviors in the past 6 months, dichotomized into never and at least one. | | |
